# Supplementary figures and images for: Accessible luminal interface of bovine rectal organoids generated from cryopreserved biopsy tissues
Source: PLoS One. 2024 Mar 21;19(3):e0301079. doi: 10.1371/journal.pone.0301079 (PMC10956885; doi:10.1371/journal.pone.0301079)

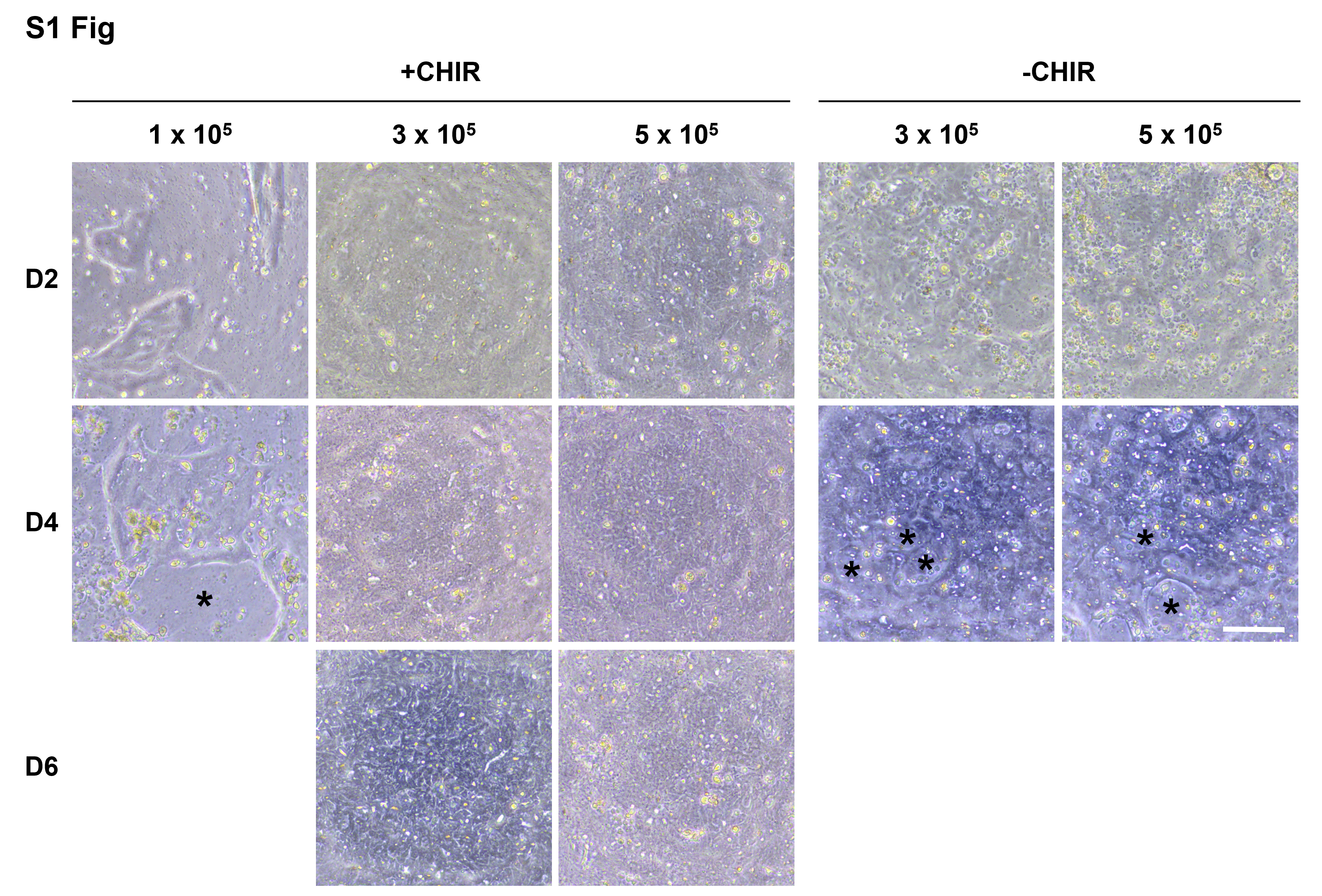

Supplement: S1 Fig — Representative phase-contrast microscopy images of 2D monolayer on Days 2, 4 and 6 (D2-6) of culture. Stable monolayer formation was achieved consistently when the cells were seeded at a density of 3–5 x 10^5^ cells per 24-well culture insert and cultured in the medium supplemented with GSK3 inhibitor (+CHIR99021). Asterisks denote areas where monolayer was not achieved or disrupted following initial monolayer formation. Bar, 100 μm. (TIF) [file pone.0301079.s001.tif]

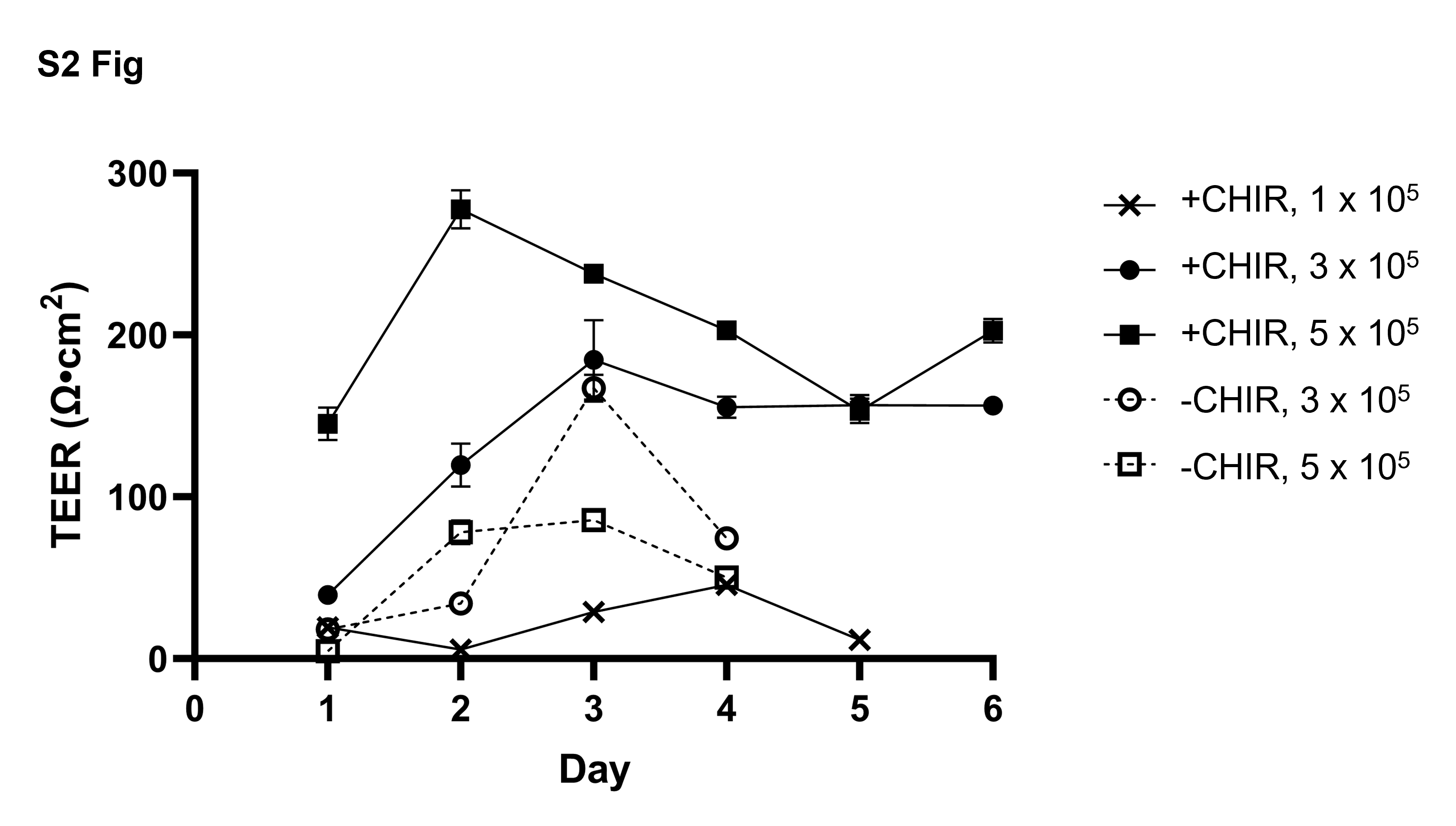

Supplement: S2 Fig — The most stable transepithelial electrical resistance (TEER) was observed when the cells were seeded at a density of 3 x10^5^ cells per 24-well culture insert and cultured in the medium supplemented with GSK3 inhibitor (+CHIR99021). The results are presented as mean ± standard error of the mean (SEM) from two technical replicates for each condition. (TIF) [file pone.0301079.s002.tif]

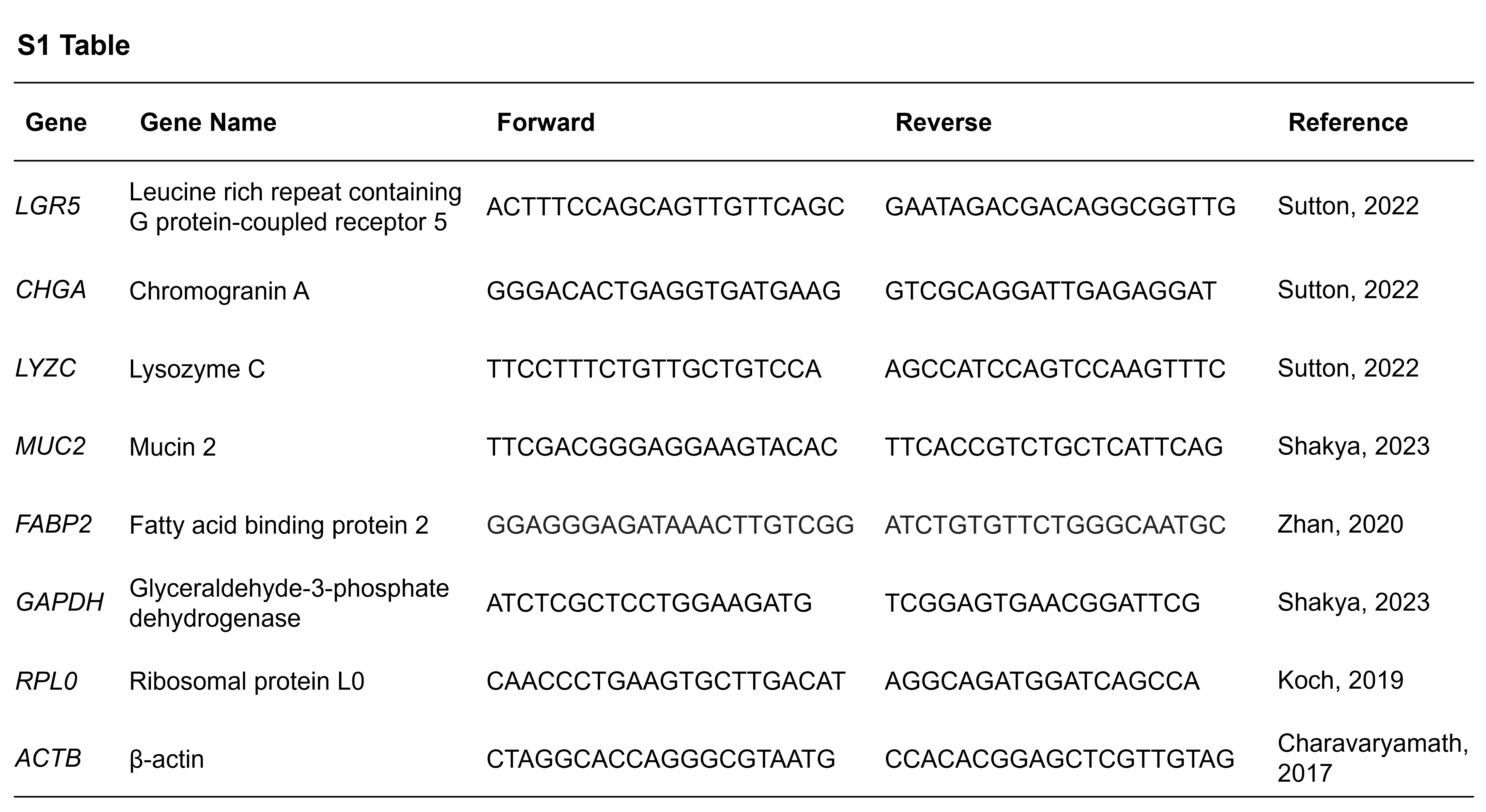

Supplement: S1 Table — Gene name, forward and reverse sequences and references are listed. (TIF) [file pone.0301079.s003.tif]

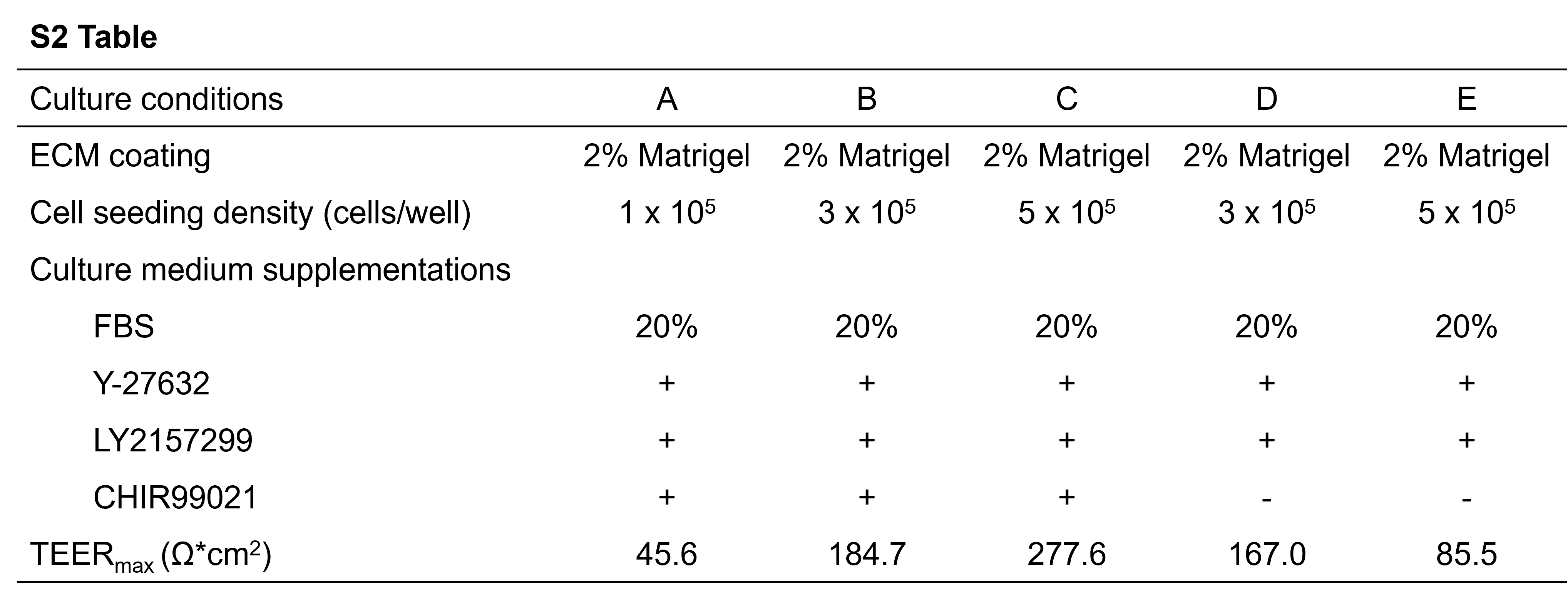

Supplement: S2 Table — Conditions of extracellular matrix (ECM) coating, cell seeding density and culture medium compositions were listed together with the maximum transepithelial electrical resistance (TEER) value that was achieved under each culture condition. (TIF) [file pone.0301079.s004.tif]
