# Supplementary material for: Accessible luminal interface of bovine rectal organoids generated from cryopreserved biopsy tissues
Source: PLoS One. 2024 Mar 21;19(3):e0301079. doi: 10.1371/journal.pone.0301079 (PMC10956885; doi:10.1371/journal.pone.0301079)
Supplement: Attachment — Submitted filename: Review Kawasaki & Ambrosini 2024.pdf [file pone.0301079.s005.pdf]

General:

The manuscript submitted by Kawasaki & Ambrosini represents an important piece of work in the field of organoid research. Their work on the characterization of bovine rectal organoids holds particular relevance for further studies related to animal welfare and addressing intestinal infections that affect these animals. The manuscript has been diligently composed, showcasing a well-defined study design and the utilization of suitable research methodologies. However, in order for it to align with the requisite standards for publication, we respectfully recommend the incorporation of the following suggestions to enhance the quality and reproducibility of the research.

Line 78 ongoing: When mentioning the collection of the tissues the authors may specify the localization of the samples, which were harvested. Were these samples harvested all at the same location or spread over a wider area of the bovine rectum?

Line 92: Is the medium used for the ´sterile wash´ the same washing medium as described above (PBS including Penicillin/streptomycin and Gentamicin)?

Line 95: The authors state the crypt isolation as "…described previously [4]". Please add another phrase stating, that the isolation is described in the following.

Line 108: There is a typo "pelted"

Line 109: Are the crypts seeded after centrifugation regardless their number? It is unlikely to expect, the all harvested material contained the same amount of crypts. Since the development form crypts to organoids and the later cultivation of the organoids is highly dependent of the number of crypts/organoids per Matrigel droplet, it is mandatory to compare cultivation methods with the same amount of crypts.

Line 112: The medium is called DMEM/F12 not DMED/F12

Lines 134-136: How large were the Matrigel droplets, which were placed, in the 48-well plates? The same size as in the 24-well plates mentioned earlier? Why the switch in plate format?

Lines 154-157: One does not measure the number of molecules but the emission as a measure for the amount of transport fluorescent dye.

Line 166: What was the solvent of BSA?

Line 175: Please state how the organoids were dissolved from the Matrigel in order to mount them on glass bottom dishes.

Line 185: Please change the writing to "marker genes"

Lines 209-214: Applying a Wilcoxon test requires testing for normal distribution of the data and used when this is not the case. Please include the statement why this test is used.

Figure 5E: The quality of the images is poor. Please change to images with better resolution to further improve the quality of the manuscript.

Lines 343-346: Please remove one of the redundant sentences.

Line 349: What does a 100% success rate mean? Just the generation of an indifferent number of organoids from crypts (quantity)? Or the successful generation from organoids from crypts regardless their numbers (quality).

Lines 396-398: If doing comparisons to other studies, please use more than one reference. Moreover, the amount of cells/cm^2 is relevant, not the total number of cells.
